# Ethylene and its crosstalk with hormonal pathways in fruit ripening: mechanisms, modulation, and commercial exploitation

**DOI:** 10.3389/fpls.2024.1475496

**Published:** 2024-11-07

**Authors:** Mohammad M. H. Tipu, Sherif M. Sherif

**Affiliations:** Virginia Tech School of Plant and Environmental Sciences, Alson H. Smith Jr. Agricultural Research and Extension Center, Winchester, VA, United States

**Keywords:** ripening, ethylene, climacteric fruit, phytohormones, signal transduction, pre-harvest drop

## Abstract

Ethylene is an important phytohormone that orchestrates a multitude of physiological and biochemical processes regulating fruit ripening, from early maturation to post-harvest. This review offers a comprehensive analysis of ethylene’s multifaceted roles in climacteric fruit ripening, characterized by a pronounced increase in ethylene production and respiration rates. It explores potential genetic and molecular mechanisms underlying ethylene’s action, focusing on key transcription factors, biosynthetic pathway genes, and signal transduction elements crucial for the expression of ripening-related genes. The varied sensitivity and dependency of ripening traits on ethylene are elucidated through studies employing genetic mutations and ethylene inhibitors such as AVG and 1-MCP. Additionally, the modulation of ripening traits by ethylene is influenced by its interaction with other phytohormones, including auxins, abscisic acid, gibberellins, jasmonates, brassinosteroids, and salicylic acid. Pre-harvest fruit drop is intricately linked to ethylene, which triggers enzyme activity in the abscission zone, leading to cell wall degradation and fruit detachment. This review also highlights the potential for applying ethylene-related knowledge in commercial contexts to enhance fruit quality, control pre-harvest drop, and extend shelf life. Future research directions are proposed, advocating for the integration of physiological, genetic, biochemical, and transcriptional insights to further elucidate ethylene’s role in fruit ripening and its interaction with other hormonal pathways.

## Highlight

- This review explores the ethylene-dependent and independent regulation of fruit ripening traits like color, firmness, starch metabolism, aroma, and fruit drop at both biochemical and molecular levels.

## Introduction

Ethylene is a crucial natural plant hormone that plays a pivotal role in regulating fruit ripening by initiating and coordinating various physiological and biochemical changes from early maturity to post-harvest. It modulates numerous ripening-related attributes such as color (Whale and Singh, 2007), firmness (Tonutti et al., 1996), total soluble solids (TSS) (Singh and Janes, 2001), aroma (Lalel et al., 2003a), respiration (Bower et al., 2002), and storage and shelf life (Xu et al., 2019). Climacteric fruits, including apples, peaches, bananas, and tomatoes, exhibit a substantial increase in ethylene production and respiration rate during ripening (Barry and Giovannoni, 2007). The autocatalytic nature of ethylene production (Yang et al., 1986) in these fruits means that once ethylene synthesis commences, it leads to a self-amplifying increase in production, thereby accelerating the ripening process. Consequently, these fruits can continue to ripen after being harvested. In contrast, non-climacteric fruits such as grapes and strawberries do not display a climacteric rise in ethylene production or respiration (Symons et al., 2012; Pérez-Llorca et al., 2019). These fruits must be harvested at full ripeness, as they do not continue to ripen post-harvest. The ripening of non-climacteric fruits is governed by plant hormones like abscisic acid (ABA), rather than ethylene (Li et al., 2022a).

**Figure 1:**
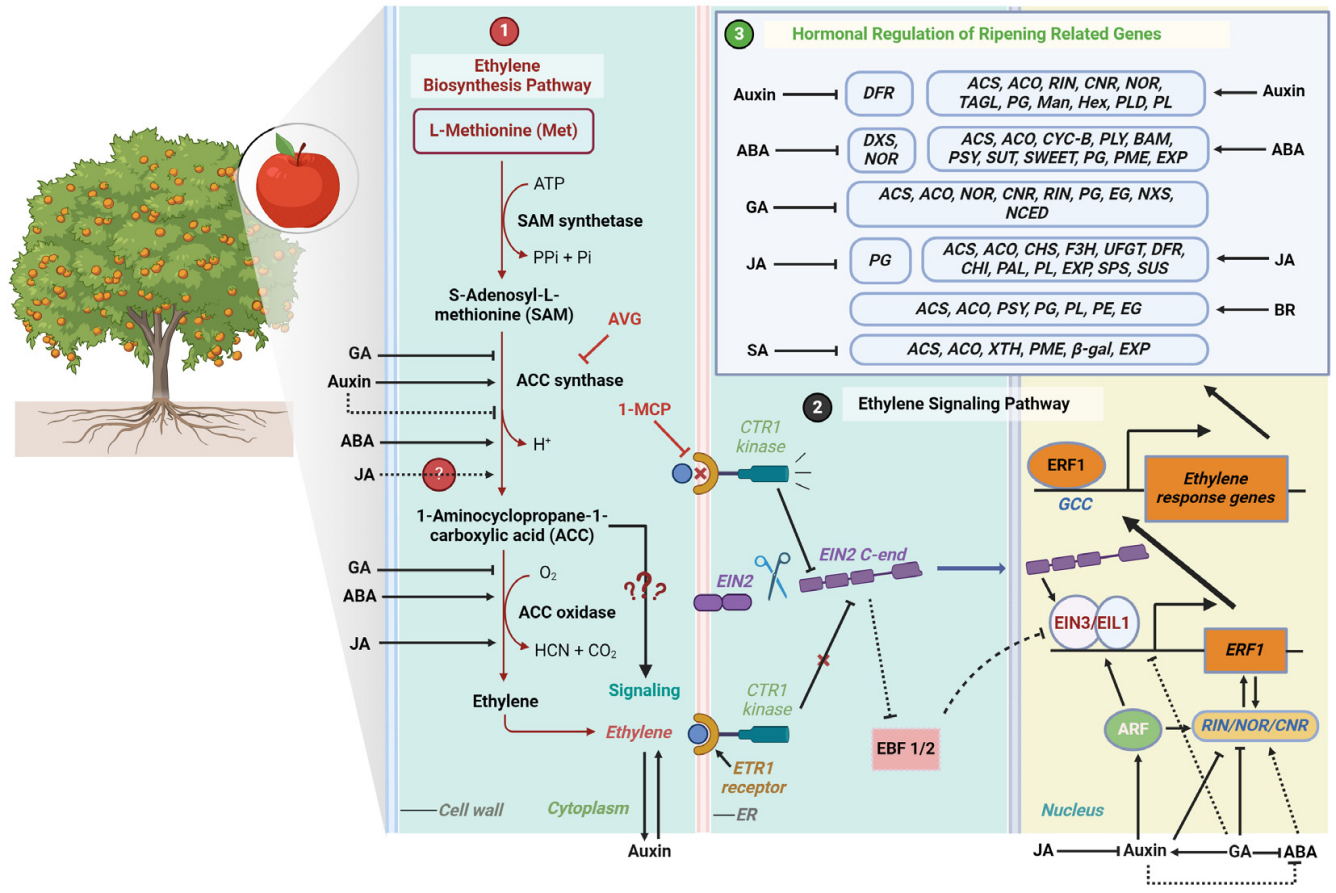
The diagram illustrates the ethylene biosynthesis and signaling pathways in plants, with an emphasis on fruit ripening and related traits modulating genes those further regulated by other hormones. 1) Ethylene biosynthesis starts with L-Methionine, which is converted to S-Adenosyl-L-methionine (SAM) by SAM synthetase using ATP. SAM is then converted to 1-Aminocyclopropane-1-carboxylic acid (ACC) by ACC synthase, a process regulated positively by abscisic acid (ABA) and auxins, and negatively by gibberellins (GA). In the next step, ACC is oxidized by ACC oxidase to produce ethylene, with its production being influenced by ABA and jasmonic acid (JA). 2) Ethylene is perceived by the ETR1 receptor on the endoplasmic reticulum (ER) membrane, initiating a signaling cascade involving CTR1 kinase and EIN2. Cleaved EIN2 translocates to the nucleus, where it stabilizes EIN3/EIL1, leading to the activation of Ethylene Response Factor 1 (ERF1). ERF1 then binds to the GCC box in ethylene response genes, regulating physiological responses such as color changes, softening, sugar accumulation, aroma production, and abscission, mediated by various hormones. ACC production can be inhibited by the application of aminoethoxyvinylglycine (AVG), which suppresses ACC synthase, while the ethylene receptor ETR1 is blocked by 1-Methylcyclopropene (1-MCP), halting ethylene signaling. 3) Other hormones like ABA, GA, Auxin, JA, BR and SA have divergent regulatory effects on ripening and related genes. A solid arrow indicates positive regulation, a solid inhibitor line indicates negative regulation, and a dotted line represents potential interaction.

Ethylene is synthesized from the amino acid methionine through a series of enzymatic reactions involving ACC (1-aminocyclopropane-1-carboxylic acid) synthase (ACS) and ACC oxidase (ACO) (**Figure 1**). ACS converts S-adenosyl-L-methionine (SAM) into ACC, which is subsequently converted to ethylene gas by ACO (Adams and Yang, 1979; Boller et al., 1979). The increased expression and activity of ACS and ACO genes result in higher ethylene production, thereby initiating and accelerating the ripening process. Ethylene can induce its own synthesis in a positive feedback loop, known as autocatalytic ethylene (Abeles et al., 2012). Ethylene perception occurs at the endoplasmic reticulum membrane, where it initiates a signal transduction cascade that elicits diverse physiological responses (Ju and Chang, 2015). The ethylene signaling pathway involves various components, including ethylene receptors such as ETR1 (Ethylene Response 1), protein kinase CTR1 (Constitutive Triple Response 1), EIN2 (Ethylene Insensitive 2), transcription factors like EIN3 (Ethylene Insensitive 3) and its homologs EILs (Ein3-Like), and ERFs (Ethylene Response Factors) (**Figure 1**). These components collectively modulate the plant’s response to ethylene and trigger downstream transcriptional reprogramming (Wang et al., 2002; Binder et al., 2004; Merchante et al., 2013). Moreover, mapping the biosynthetic and signal transduction pathways of ethylene provides a critical framework for understanding the multi-layered and complex regulation of ripening traits. This pathway mapping enables researchers to uncover points of interaction and regulation, which are essential for both basic scientific understanding and the development of applied strategies particularly in pre- and post-harvest fruit management.

The role of ethylene in the development of various fruit ripening traits has been elucidated not only through ethylene application and quantification but also through genetic mutations and the use of ethylene inhibitors. Key transcription factors, such as Ripening Inhibitor (RIN), Colorless Non-Ripening (CNR), and Non-Ripening (NOR), play crucial roles in fruit ripening. These transcription factors regulate the expression of genes involved in ripening, and their activity is influenced by ethylene signaling (Liu et al., 2016; Mou et al., 2016). For example, studies using RIN tomato mutants demonstrated that these mutated fruits do not exhibit the typical climacteric rise in ethylene and show delayed ripening (Herner and Sink, 1973). Ethylene can initiate the ripening of mature green fruit and upregulate RIN expression (Li et al., 2020), but RIN-deficient fruit never fully ripens, even with exogenous ethylene, indicating that both RIN and ethylene are required for complete ripening. On the other hand, ethylene inhibitors such as aminoethoxyvinylglycine (AVG) and 1-methylcyclopropene (1-MCP) play negative roles in modulating the ripening process of climacteric fruits. AVG directly inhibits ACC synthase activity, resulting in lower ethylene production in treated fruits (Amrhein and Wenker, 1979; Yu et al., 1979; Yang and Hoffman, 1984) (**Figure 1**). In contrast, 1-MCP does not directly inhibit ethylene biosynthesis but prevents the action of ethylene by binding to its receptors (Sisler and Serek, 1997; Sisler et al., 1999; Blankenship and Dole, 2003) (**Figure 1**). Consequently, while ethylene may still be produced in 1-MCP-treated fruits, its effects on ripening are nullified.

The influence of ethylene on various ripening-related traits frequently suggests the hypothesis that ethylene-dependent characteristics may cluster or segregate in a distinct manner during the ripening process. These traits typically exhibit a coordinated sequence of changes, although the degree and nature of their clustering can vary. In the early ripening phase, increased ethylene synthesis initiates changes in cell wall structure, beginning with the solubilization of pectin (Rose et al., 1998; Ponce et al., 2010). Simultaneously, significant chlorophyll degradation and enhanced pigment synthesis result in visible color changes (Whale and Singh, 2007; Su et al., 2015; Chen et al., 2022). During the mid-ripening stage, further breakdown of cell wall components leads to noticeable fruit softening and at the same time aroma volatile compounds starts to synthesize (El Hadi et al., 2013; Tucker et al., 2017; Wang et al., 2018b). Later, cell wall degradation reaches its peak, rendering the fruit very soft, while the characteristic aroma fully develops, and starches are completely converted to sugars (Farcuh et al., 2018), resulting in the fruit’s sweetest phase.

However, ethylene-dependent traits do not always segregate uniformly; they can exhibit distinct patterns influenced by the interplay of other hormones, genetic factors, environmental conditions, and developmental stages (Stepanova et al., 2005; Barry and Giovannoni, 2007; Klee and Giovannoni, 2011; Sobolev et al., 2014; Liu et al., 2020). For instance, in seminal studies by Pech et al. (2008) and Johnston et al. (2009), the authors explored the effects of silencing the ACO gene on ethylene production and ripening in melon and apple, respectively. By reducing ethylene synthesis, they could examine ripening traits without ethylene interference. Both investigations concluded that individual ripening traits, such as color change, volatile production, and softening, exhibit varying sensitivities to and dependencies on ethylene (Pech et al., 2008; Johnston et al., 2009). Specifically, Johnston et al. (2009) demonstrated that early ripening traits, like starch conversion to sugars, showed low ethylene dependency but high sensitivity to low ethylene concentrations. In contrast, traits such as flesh softening and volatile production displayed high ethylene dependency but require higher ethylene concentrations to elicit responses. Not only can ethylene-dependent ripening traits segregate non-uniformly, but research conducted on tomatoes (Murray et al., 1993), bananas (Golding et al., 1998), and melons (Hadfield et al., 2000) using genetic mutations in ethylene biosynthesis and employing ethylene inhibitors has also demonstrated that both ethylene-dependent and ethylene-independent pathways exist for the regulation of ripening traits in climacteric fruits.

In this review, we aim to provide a comprehensive analysis of the multifaceted roles that ethylene plays during the ripening of climacteric fruits. Emphasis will be placed on the diverse fruit ripening traits, elucidating their dependence or independence on ethylene, and exploring the roles of other plant hormones in the ripening process and their potential interactions. We will examine these elements from physiological, genetic, biochemical, and transcriptional perspectives, highlighting the utility of genetic mutations and ethylene inhibitors in addressing key questions. Finally, this article will discuss how this knowledge can be translated into commercial applications and explore future perspectives in this field of research.

## Ethylene regulation of fruit ripening traits from molecular, biochemical and physiological perspectives

The correlation between ethylene levels and ripening traits is a fundamental aspect of fruit physiology and post-harvest management. Understanding the biochemical correlation between ethylene and ripening traits involves delving into the molecular and biochemical mechanisms underlying this process. This section discusses how ethylene affects different ripening traits.

### Ethylene regulation of fruit coloration

The alteration in fruit coloration is a pivotal indicator of ripeness, predominantly governed by heightened ethylene production in various climacteric fruits. In exemplars such as apples and tomatoes, ethylene production intensifies red pigmentation, whereas in avocados, it transits the green hue to a purple or black due to the augmented concentration of anthocyanins, particularly cyanidin 3-O-glucoside (Dong et al., 1995; Cox et al., 2004; Whale and Singh, 2007). In mangoes, ethylene facilitates chlorophyll degradation by accelerating the activities of chlorophyllase and Mg-dechelatase, and it enhances anthocyanin content by regulating a series of enzymes including phenylalanine ammonia-lyase (PAL), chalcone isomerase (CHI), dihydroflavonol 4-reductase (DFR), and UDP-glucose: flavonoid 3-O-glucosyl transferase (UFGT) (Chen et al., 2022). This leads to the manifestation of the characteristic red, yellow, or orange colors in ripe mangoes. Conversely, in fruits such as plums, skin color development is not directly influenced by ethylene; rather, ethylene acts as a catalyst, expediting and synchronizing pigment production and chlorophyll degradation (Abdi et al., 1998). The intensity and timing of these color changes are modulated by ethylene signaling associated with system-2 ethylene, known for its autocatalytic nature (Huang et al., 2022).

At the transcriptional level, ethylene significantly influences the expression of genes responsible for the biochemical transformations that lead to color change in fruits. In apples, ethylene enhances anthocyanin biosynthesis and fruit coloration by inducing the expression of MYB transcription factors. Specifically, *MdMYB1* is crucial in regulating anthocyanin biosynthesis and accumulation. MdEIL1 directly activates *MdMYB1* expression, which in turn promotes anthocyanin accumulation (An et al., 2018). MdMYB1 also activates *MdERF3*, further enhancing ethylene production (An et al., 2018). This regulatory loop is modulated by MdMYB17, which represses *MdMYB1* and *MdEIL1* expression (Wang et al., 2022b). Additionally, ethylene-activated MdPUB24 ubiquitinates MdBEL7, thereby upregulating the expression of chlorophyll catabolic genes such as *MdCLH* (Chlorophyllase), *MdPPH2* (Pheophytinase), and *MdRCCR2* (Red Chl Catabolite Reductase), leading to chlorophyll degradation (Wei et al., 2021). The never-ripe (*Nr*) tomato mutant, which is insensitive to ethylene due to a mutation in the ethylene receptor gene (ETR1), provides further evidence of ethylene’s role in color change, as both coloration and softening are impaired in this mutant (Wilkinson et al., 1995).

Investigations utilizing ethylene inhibitors, such as AVG and 1-MCP, provide robust evidence on ethylene’s regulation of chlorophyll degradation and the synthesis of pigments like carotenoids and anthocyanins in various fruits (Bangerth, 1978; Clayton et al., 2000; Feng et al., 2000; Phan-Thien et al., 2004; Whale et al., 2008; Lee et al., 2010; Lv et al., 2020; Zhang et al., 2020). For instance, 1-MCP treatment in Chinese pears delays chlorophyll degradation by inhibiting ethylene production and suppressing the expression of genes such as Pheophorbide A Oxygenase (*PAO*), Non-Yellow Coloring (*NYC*), Nyc1-Like (*NOL*), and Stay-Green1 (*SGR1*) (Cheng et al., 2012). Similarly, in plums, 1-MCP delays anthocyanin accumulation by suppressing the expression of biosynthesis-related genes, including Phenylalanine Ammonia-Lyase (*PsPAL*), Chalcone Synthase (*PsCHS*), Chalcone Isomerase (*PsCHI*), Flavanone 3-Hydroxylase (*PsF3H*), Dihydroflavonol 4-Reductase (*PsDFR*), Anthocyanidin Synthase (*PsANS*), Udp-Glucose: Flavonoid 3-O-Glucosyl Transferase (*PsUFGT*), and the transcription factor *PsMYB10* (Xu et al., 2020). Moreover, AVG delays red coloration in apples, with its effect being dose-dependent and influenced by climatic conditions (Greene, 2005; Yildiz et al., 2012; Liu et al., 2022). Collectively, these findings underscore ethylene’s critical role as a regulator of color change in climacteric fruits, orchestrating complex biochemical pathways and gene expression mechanisms. Genetic mutations, biochemical analyses, gene expression studies, and the application of ethylene inhibitors all illuminate the multifaceted role of ethylene in this essential ripening trait.

### Ethylene regulation of fruit softening and firmness

Ethylene plays a crucial role in fruit softening and firmness by activating cell wall-degrading enzymes, such as polygalacturonase, which hydrolyze pectin in the cell walls, leading to fruit softening. For instance, the stony hard (*hd*) peach mutant, which lacks ethylene production likely due to allelic variation within the first intron of the *PpYUC11*-like gene (a YUCCA-like auxin-biosynthesis gene), maintains firm flesh but softens when treated with exogenous ethylene (Hayama et al., 2006; Cirilli et al., 2018). Similarly, kiwifruit lines with suppressed ethylene production due to the knockdown of the *ACO1* gene retain their firmness but soften upon exogenous ethylene treatment (Atkinson et al., 2011). In tomatoes, an abnormally ripening ‘Alcobaca’ mutant that carries a third allele at the *nor* locus, characterized by reduced ethylene production, shows increased storability and slower softening during storage (Lobo et al., 1984; Mutschler, 1984; Lu et al., 1993). Consequently, ethylene negatively impacts fruit firmness and shelf life, with ethylene-suppressed fruits being firmer and having a longer shelf life (Dandekar et al., 2004).

Ethylene also regulates enzymes responsible for the catabolism of cellulose and pectin, resulting in the dismantling of polysaccharide networks (Prasanna et al., 2007). Various cell wall-related genes, such as Polygalacturonase1 (*PG1*), β-Galactosidase1 (*BGAL1*), Xyloglucan Endotransglucosylase/hydrolase1/2 (*XTH1/2*), and Expansins (*EXP*s), exhibit differential sensitivities to and dependencies on ethylene (Johnston et al., 2009; Ireland et al., 2014). For example, the expression of *PG* genes, which are responsible for cell wall degradation and fruit softening, is regulated by both ethylene-dependent and ethylene-independent factors (Oeller et al., 1991; Pech et al., 2008).

Ethylene biosynthetic inhibitors, such as AVG, delay softening in various climacteric fruits, including peaches (Hayama et al., 2008), apples (Kon et al., 2023), pears (He et al., 2023), and nectarines (McGlasson et al., 2005). Conversely, exogenous ethylene promotes the expression of cell wall-degrading genes, particularly *PG* in apples (Tacken et al., 2010), and activity of enzymes like PG, pectin esterase (PE), and cellulase in guavas (Abu-Goukh and Bashir, 2003), which reduce firmness. In apple and peach, AVG-treated fruits retain firmness longer by reducing PG activity in fruit abscission zones (Wang and Dilley, 2001; Belding and Lokaj, 2002; Zhu et al., 2008). Similarly, apple fruits treated with 1-MCP remain firmer during storage and transportation (Watkins et al., 2000; Win et al., 2019, 2021). Additionally, in papaya, 1-MCP treatment delays ripening and maintain normal characteristics when applied for a short-term (Zhu et al., 2019).

### Ethylene’s influence on sugar metabolism and accumulation

The role of ethylene in sugar metabolism and accumulation is multifaceted and varies among different types of fruits, exhibiting diverse regulatory mechanisms. In Japanese plums, banana and blueberry, ethylene significantly influences sugar metabolism by reducing sucrose catabolism and promoting sucrose biosynthesis (Farcuh et al., 2018; Cordenunsi-Lysenko et al., 2019; Wang et al., 2020b). Conversely, antisense *ACO* melons demonstrate higher soluble solids content despite a lower ripeness index (Martínez-Madrid et al., 2002). These melons exhibit suppressed ethylene biosynthesis, show reduced ethylene production by up to 97.7% compared to wild-type fruit (Martínez-Madrid et al., 2002). Similarly, in tomatoes, the metabolism of citrate, malate, and starch operates independently of ethylene (Jeffery et al., 1984). In kiwifruit, low temperatures during storage can stimulate sugar accumulation without triggering ethylene and ethylene-dependent aroma volatiles, indicating that ethylene signaling is inactive during this process (Mitalo et al., 2019). The transcript levels of the MADS-box transcription factor AcMADS2, which regulates the starch degradation gene β-Amylase2 (*Acβ-AMY2*) and the sucrose metabolism gene Invertase (*AcINV3-1*), increased only in response to low temperature (5°C). Treatment with propylene, an ethylene analog, did not affect these transcript levels (Mitalo et al., 2019).

Ethylene plays a critical role in the conversion of starches to sugars in fruits, whereas ethylene inhibitors tend to delay this process, resulting in higher starch content and a slower increase in sugars (Yuan and Carbaugh, 2007; Yildiz et al., 2012; Brackmann et al., 2015; Malladi et al., 2023). Specifically, amylase activity in AVG-treated apples is reduced during the initial stages of starch mobilization, while starch phosphorylase activity significantly increases during the later stages and remains unaffected (Silverman et al., 2004). Similarly, 1-MCP-treated apples experience slower starch breakdown, enabling delayed harvesting without compromising quality (Tomala et al., 2020). In bananas, the levels of β-amylase protein, responsible for hydrolyzing the α-1,4-glucosidic linkages in starch, are nearly undetectable in 1-MCP-treated fruit (do Nascimento et al., 2006). Conversely, AVG does not alter the levels of amylopectin, fructose, malate, ascorbate, and citrate in apples (Silverman et al., 2004), while 1-MCP increases the activity of α-1,4-glucan-phosphorylase in bananas. This enzyme, essential for starch synthesis and degradation, suggests that the absence of ethylene perception positively affects phosphorylase activity (Mainardi et al., 2006). Collectively, these findings indicate that ethylene is not the sole regulator of carbohydrate metabolism during fruit ripening; other pathways may also play a significant role in starch degradation and sugar accumulation.

### Ethylene regulation of aroma development in fruits

Aroma development is a critical trait influenced by ethylene and intricately linked to ethylene levels. Elevated ethylene production enhances the synthesis of aroma volatile compounds (Zhang and Jia, 2005). Post-harvest applications of acetaldehyde and ethanol, both associated with ethylene, improve the sensory quality of tomatoes and pears (Paz et al., 1982). Further research confirms that ethylene-dependent processes significantly contribute to aroma production, as observed in Charentais cantaloupe melons (Pech et al., 2001; Bauchot et al., 2002). In these melons, reduced ethylene production via ACO antisense mRNA expression delays ripening and decreases total volatile composition (Bauchot et al., 1998). Similarly, genetic modifications to suppress ethylene biosynthesis by silencing *ACS* and *ACO* genes in apples result in substantial reductions in aroma volatile esters (Dandekar et al., 2004). In Greensleeves apples with high suppression of ethylene biosynthesis, Defilippi et al. (2005) found that levels of aroma volatile-related enzymes, including alcohol acyltransferase (AAT), alcohol dehydrogenase (ADH), and lipoxygenase (LOX), along with amino acids and fatty acids as aroma volatile precursors, varied between peel and flesh tissues. Generally, volatile production, enzyme activity levels, and precursor availability were higher in the peel than in the flesh and were differentially affected by ethylene regulation. AAT enzyme activity showed a clear pattern consistent with ethylene regulation. However, it should be noted that some upstream steps in the aroma biosynthetic pathway, such as the production of alcohols and aldehydes, remained unaffected in the study by Dandekar et al. (2004), indicating that ethylene does not control every aspect of flavor production. Additionally, Tsantili and Knee (1991) found that storing apples in low-ethylene conditions for extended periods did not impair the synthesis of aroma volatiles, suggesting that ethylene may not be the sole contributor to the production of these compounds.

Given that ethylene promotes the biosynthesis of aroma volatile compounds, ethylene inhibitors can significantly impact this process. Fruits treated with 1-MCP demonstrated inhibited production of volatile compounds such as 2-methylbutyl acetate, butyl acetate, hexyl acetate, and butanol, which contribute to flavor in apples (Fan and Mattheis, 2001; Marin et al., 2009). At the transcriptional level, ethylene treatment upregulates the expression of genes involved in volatile biosynthesis in apple fruit, including branched-chain amino acid aminotransferase (BCAT), aromatic amino acid aminotransferase (ArAT), amino acid decarboxylases (AADC), and various enzymes related to fatty acid synthesis and metabolism. Conversely, treatment with 1-MCP generally produces opposite effects on these genes, providing further evidence that their regulation is ethylene-dependent (Yang et al., 2016).

### Ethylene regulation of respiration and shelf life in climacteric fruits

Changes in respiration rate and ethylene production in climacteric fruits follow a typical pattern, with the peak of ethylene production coinciding with an increase in respiration (Abbas and Fandi, 2002). The interaction between ethylene and respiration significantly impacts the storage and shelf life of fruits. High ethylene levels induced by substances like ethephon can accelerate the respiration rate and ethylene production during ripening (Lalel et al., 2003a). Maintaining lower ethylene levels can lead to more uniform ripening in fruits such as plums (Rozo-Romero et al., 2015). However, in mangoes, research has shown that respiration can remain relatively constant regardless of the absence or low levels (0.005 μL/L) of endogenous ethylene when treated with short-term ultraviolet-C light (180-280 nm) during storage (Pristijono et al., 2019). Although the molecular links between ethylene and respiration have not been extensively examined, recent studies suggest that the alternative oxidase (AOX) respiratory pathway may play a crucial role in mediating the crosstalk between ethylene response, carbon metabolism, ATP production, and reactive oxygen species (ROS) signaling during climacteric ripening (Hewitt and Dhingra, 2020). Furthermore, in tomatoes, the relationship between respiration rate and ethylene production varies among different fruit sizes and cultivars, with small-fruited types generally exhibiting higher respiration rates and ethylene production compared to medium and large-fruited varieties (Ustun et al., 2023).

Controlled ethylene levels can reduce respiration rates, further delaying starch breakdown, resulting in firmer and less sweet fruits during storage, thereby extending shelf life (Thammawong and Arakawa, 2007). Paull and Chen (1983) found a positive correlation between polygalacturonase and xylanase activity and respiration in papaya. Inhibiting ethylene biosynthesis with compounds like AVG can reduce the climacteric rise in respiration associated with ripening, thereby slowing down the ripening process (Whale et al., 2008; Çetinbaş et al., 2012). Similarly, post-harvest treatments such as controlled atmosphere storage in low O_2_ concentration (0.5-1.0%) and the use of ethylene inhibitors like 1-MCP are employed to manipulate ethylene levels and prolong the shelf life of pears (Chen et al., 1981; Argenta et al., 2003). In apples, 1-MCP effectively delays the climacteric peak of respiration and ethylene production (Lv et al., 2020). Additionally, the application of chlorine dioxide (ClO_2_) effectively reduces respiration rates in ‘Hami’ melon fruit, combined with a downregulation of ethylene-biosynthesis genes such as *CmACS2*, *CmACO1*, and *CmACO3* (Guo et al., 2013). These studies indicate that controlling ethylene levels with inhibitors can be effective in extending the shelf life of fruits by reducing respiration rates.

## Roles of other plant hormones affecting ripening

Ethylene has historically been recognized as the principal regulator of ripening in climacteric fruits. However, over the past two decades, there has been a significant accumulation of knowledge regarding the intricate interplay between ethylene and other phytohormones during the ripening process. This section delves into the influence of additional plant hormones such as auxin, ABA, gibberellins (GA), and jasmonates (JA) on various ripening characteristics.

### Auxins

Auxin, particularly indole-3-acetic acid (IAA), plays a pivotal role in the initial stages of fruit development. Auxin biosynthesis and signaling pathways were reviewed by Gomes and Scortecci (2021). In climacteric fruits such as apples and tomatoes, auxin is essential for early fruit development and fruit set (Shin et al., 2016; Zhang et al., 2021). During the transition to ripening, auxin levels rise in climacteric fruits, thereby initiating the ripening processes (Trainotti et al., 2007). By the late stages of ripening, auxin concentrations diminish, facilitating the complete maturation and senescence of the fruit, as observed in peaches (Trainotti et al., 2007). Auxin is also known to enhance ethylene production in numerous horticultural crops. Indeed, the promoter regions of several auxin response factors (ARFs) contain cis-regulatory elements for both auxin and ethylene, indicating that these ARFs may be regulated by both hormones (Muday et al., 2012; Zouine et al., 2014). Recent research has also shown that auxin induces ethylene biosynthesis in apple fruits by activating the expression of the auxin response factor *MdARF5* (**Figure 1**). MdARF5 binds to the promoter of *MdERF2*, which encodes a transcription factor in the ethylene signaling pathway, as well as to the promoters of two ACC synthase genes, *MdACS3a* and *MdACS1*, and an ACC oxidase gene, *MdACO1* (Yue et al., 2020). Similarly, in tomatoes, Auxin Response Factor2 (SlARF2) functions as a positive regulator of ripening, despite being a transcriptional repressor (Hao, 2014). Fruits with reduced expression of *SlARF2* produce less climacteric ethylene and exhibit down-regulation of key ripening genes such as *RIN*, *CNR*, *NOR*, and *TAGL1* (Tomato Agamous-Like 1) (Hao et al., 2015). Mechanistically, the ethylene response factor SlERF.D7 directly targets and positively regulates *SlARF2A* and *SlARF2B*, thereby integrating auxin and ethylene signaling pathways in the control of ripening (Gambhir et al., 2022). Additionally, silencing Auxin Response Factor4 (*ARF4*), which is strongly expressed in tomato pericarp, resulted in increased accumulation of starch and chlorophyll content. This finding suggests that auxin signaling is involved in the regulation of chloroplastic activity and sugar metabolism in the fruit (Jones et al., 2002; Sagar et al., 2013). The elevated starch content in developing fruits of *SlARF4* down-regulated lines correlated with the up-regulation of genes and enzyme activities involved in starch biosynthesis, implying negative regulation by SlARF4 (Sagar et al., 2013). Notably, the application of exogenous auxin has been found to influence the expression levels of genes such as *PpPG2*, thereby reducing fruit firmness in peach fruits (Tatsuki et al., 2013).

Auxin has also been observed to exert negative effects on various ripening traits. For example, auxins tend to suppress the climacteric rise in respiration rate by downregulating numerous genes involved in the tricarboxylic acid (TCA) cycle in tomatoes (Frenkel and Dyck, 1973; Li et al., 2016; Yue et al., 2020). Additionally, auxin inhibits the synthesis of pigments such as anthocyanin in apples, with the degree of inhibition increasing with higher doses (Ji et al., 2015). (Wang et al., 2018a) further elucidated that the auxin response factor MdARF13 acts as a negative regulator of anthocyanin biosynthesis via the Aux/IAA–ARF signaling pathway, directly binding to the promoter of *MdDFR*, a structural gene in the anthocyanin biosynthetic pathway in apples. Similarly, Wu et al. (2018a) discovered that exogenous auxin negatively impacts the accumulation of aroma volatiles during tomato fruit ripening. In a separate study on Japanese plums (*P. salicina L.*), it was found that auxin advances ripening independently of ethylene action by modulating cell wall genes encoding α-Mannosidase (*α-Man*), β-d-N-Acetylhexosaminidase (*β-Hex*), Phospholipase d-α (*PLD*), and Pectate Lyase (*PL*) (El-Sharkawy et al., 2016) (**Figure 1**). These studies collectively suggest that auxin’s role in regulating the ripening of climacteric fruits can be mediated through the induction of ethylene production, but it may also function independently to regulate specific ripening traits, particularly during the later stages of fruit ripening.

### Abscisic acid

Abscisic acid (ABA) has been demonstrated to promote fruit ripening, enhancing color change and accelerating sugar accumulation in fruits such as date palm and banana (Jiang et al., 2000; Elbar et al., 2023). In kiwifruit, ABA treatment increases the production of esters and other aroma compounds by enhancing the activity of related enzymes, particularly alcohol acyltransferase (AAT), branched amino acid transaminase (BCAT), and hydroperoxide lyase (HPL) (Han et al., 2022). ABA accelerates both ethylene production and respiration in bananas (Jiang et al., 2000), and the application of ABA accelerates ethylene biosynthesis by promoting the activities of ethylene biosynthesis enzymes, ACC synthase and ACC oxidase, and the accumulation of ACC in mango and tomato (Zhang et al., 2009; Zaharah et al., 2013) (**Figure 1**). At the genetic level, overexpressing the persimmon gene *DkBG1*, which encodes β-glucosidase 1 responsible for cleaving ABA-glucose esters to release active ABA, in tomato plants hastened fruit ripening by 3-4 days (Liang et al., 2020). Furthermore, these transgenic tomatoes exhibited modified expression of the ripening regulator gene, *NOR*, and its downstream targets, resulting in noticeable changes in fruit coloration by downregulating the expression of 1-Deoxy-D-Xylulose-5-Phosphate Synthase (*DXS1*) and Phytoene Synthase (*PSY1*), while significantly upregulating the expression of lycopene β-Cyclase (*CYC-B*) (Liang et al., 2020) (**Figure 1**). The interaction between ethylene and ABA during tomato fruit ripening is supported by the finding that SlAREB1, an ABA-responsive transcription factor, directly activates NOR (Mou et al., 2018). Additionally, transient overexpression of *SlAREB1* in tomato fruits results in increased levels of several downstream ethylene biosynthetic genes, such as *SlACS2*, *SlACS4*, and *SlACO1*, thereby linking ABA signaling to ethylene production and ripening (Mou et al., 2018). Abscisic acid (ABA) biosynthesis and signaling mechanisms were reviewed by Hauser et al. (2011).

The role of ABA in ripening is contingent upon the induction of various transcription factors and is frequently modulated by interactions with inhibitors. In mango, (Wu et al., 2022) discovered that ABA is closely linked to ripening, where ABA-responsive transcription factors such as long hypocotyl5 (MiHY5) directly regulate ripening-related genes like Pectate Lyase (*MiPLY8*), Beta-Amylase (*MiBAM9*), and Phytoene Synthase (*MiPSY*) (**Figure 1**). Another study revealed that the ABA-responsive transcription factor MdAREB2 directly activates the expression of sugar transporter genes, specifically Tonoplast Monosaccharide Transporters *MdTMT1* and Sucrose Transporter *MdSUT2*, to enhance soluble sugar accumulation in apple (Ma et al., 2017). Additionally, the transcription factor MdWRKY9, induced by ABA, positively regulates the expression of the apple SWEET gene *MdSWEET9b*, a sucrose transporter gene, thereby promoting sugar accumulation in apple fruits (Zhang et al., 2023a).

ABA also influences fruit firmness by differentially affecting the expression of cell wall-related genes such as *PG*, *PME*, *EXP1*, and *EXP2*, thereby modulating the ripening process depending on the developmental stage (Soto et al., 2013) (**Figure 1**). In tomatoes, the key enzyme 9-cis-epoxycarotenoid dioxygenase1 (LeNCED1), which initiates ABA biosynthesis at the onset of fruit ripening, acts as a potential inducer, and treatment with the ABA inhibitor nordihydroguaiaretic acid delayed fruit ripening and softening (Zhang et al., 2009). Interestingly, the ABA-induced tomato ripening was not observed in fruits treated with 1-MCP, indicating that ABA’s stimulation of ripening progress is at least partially dependent on ethylene signaling (Mou et al., 2016). Overall, most studies on the role of ABA in the ripening of climacteric fruits suggest that ABA acts as a positive regulator of many ripening traits, primarily through its interaction with ethylene.

### Gibberellins

Gibberellins (GA) play a negative role in fruit ripening, delaying the ripening process by inhibiting ethylene production (Chen et al., 2020) and regulating the expression of ripening related genes *NOR*, *CNR* and *RIN* (Li et al., 2019) (**Figure 1**). GA biosynthesis and its regulation were reviewed by Hedden and Thomas (2012). In mango and nectarine, GA_3_ is responsible for the delay in chlorophyll degradation and decreases the total carotenoid content including anthocyanin during ripening and storage (Khader, 1991; Zilkah et al., 1997). Gibberellins can also delay the increase in TSS by prolonging the fruit development stage. In banana, GA_3_ slows down the onset of starch degradation by delaying starch phosphorylase activity and delays sucrose accumulation affecting sucrose phosphate synthase activity by at least 2 days (da Mota et al., 2002; Rossetto et al., 2003). GA_3_ also delays cell wall modification related to fruit softening. These changes involve inhibition of the breakdown of the middle lamella, separation of the plasmalemma from the cell wall, increased structural integrity of the primary cell wall, decreased solubilization of pectic polymers, loss of neutral sugars (arabinose and galactose), and lower activities of enzymes PG and endo-1,4-β-glucanase (EG) (Ben-Arie et al., 1996). Moreover, gibberellins delay the climacteric rise by maintaining a lower respiration rate (Nakano et al., 1997).

The role of gibberellins in ripening further explored by Wu et al. (2024) by overexpressing the gibberellin synthesis gene *SlGA3ox2* specifically in fruit tissues. The transgenic tomato fruits exhibited delayed fruit ripening, whereas treatment with the GA biosynthesis inhibitor paclobutrazol (PAC) accelerated their fruit ripening (Wu et al., 2024). The involvement of GA in the repression of fruit ripening may occur through the modulation of auxin metabolism and/or signal transduction since many genes related to auxin biosynthesis or signaling pathways were altered in GA-treated fruits (Wu et al., 2024). This hypothesis was previously investigated by Park and Malka (2022) where they showed that GA delays metabolic shifts during fruit ripening by inducing auxin signaling in tomato. However, the effect of auxin on GA-responses seems to be species/organ/developmental stage-dependent (Sun and Gubler, 2004). Moreover, in tomato fruits, GAs downregulate Neoxanthine Synthase (*NXS*) and 9-Cis-Epoxycarotenoid Dioxygenase (*NCED*), *ACS* and *ACO* genes encoding the biosynthesis of ABA and ethylene (Obroucheva, 2014) further underscored it’s negative role in ripening (**Figure 1**).

### Jasmonates

Jasmonates (JAs), key phytohormones associated with stress responses, exhibit a multifaceted role in fruit ripening, acting as either promoters or inhibitors depending on the context and fruit type. JAs are known to activate ethylene biosynthesis genes, thereby enhancing the climacteric rise in ethylene production (Kondo et al., 2007; Li et al., 2017). The application of n-propyl dihydrojasmonate (PDJ), a JA derivative, has been shown to increase the expression of *ACS1* and *ACO1* genes in the skin of tomatoes, apples, and pears at the pre-climacteric stage, while decreasing *ACS1* mRNA accumulation during the climacteric stage (Fan et al., 1998; Kondo et al., 2007; Kondo, 2010). Additionally, exogenous JAs promote color change in a concentration- and time-dependent manner during the onset of ripening in apples. For ‘Cripps Pink’ apples, a single pre-harvest spray of methyl jasmonate (MeJA), another derivative of JA, at the optimal time is more effective than multiple applications in enhancing red blush and export-grade fruit quality (Shafiq et al., 2013). Similarly, PDJ application in ‘Gala’ and ‘Braeburn’ apples shows optimal effects on coloration when applied about two weeks before harvest (Atay, 2015). JA may act also as an inhibitor. While auxin acts as a positive regulator of ripening, exogenous JA generally down-regulated genes associated with auxin signal transduction in apples (Feng et al., 2017). A review by Han (2017) provides an in-depth examination of the biosynthesis and signaling pathways of JAs.

At the transcriptional level, MeJA enhances the expression of several structural genes involved in anthocyanin biosynthesis, including *PpCHS*, *PpUFGT*, *PpF3H*, *PpDFR*, *PpCHI*, and *PpPAL*, as well as the transcription factor *PpMYB10* during peach fruit ripening (Wei et al., 2017) (**Figure 1**). In red Chinese pear fruits, JAs have been found to induce anthocyanin accumulation through the regulation of transcription factors such as *PpMYB10*, *PpMYB114*, and a basic helix-loop-helix (*bHLH*), independent of ethylene (Ni et al., 2020).

JAs also regulate other fruit quality attributes such as fruit firmness, soluble solids content, flavor, and aroma. In peaches, MeJA increases the expression of cell wall-associated genes, such as *PpPL*, *PpEXP1*, and *Ppcel1* (Cellulase), while decreasing the expression and enzymatic activity of the *PpPG* gene (Ziosi et al., 2008a; Wei et al., 2017) (**Figure 1**). Conversely, in apples, the expression of both *MdPL* and *MdPG* was significantly downregulated when treated with MeJA, leading to prolonged firmness (Ding et al., 2024). Jasmonates influence TSS by modulating sugar metabolism and transport. JA-treated Japanese plums and tomatoes exhibited higher TSS levels, total antioxidant activity, and total phenolics (Khan and Singh, 2007; Kucuker et al., 2014; Tao et al., 2022). In tomatoes, JA treatment increased the expression of genes related to sugar metabolism, such as Amylase, Sucrose Phosphate Synthase (*SPS*), and Sucrose Synthase (*SUS*), thereby enhancing sugar accumulation at optimal concentrations (Li et al., 2022b). The application of MeJA resulted in higher levels of fatty acids and a notable increase in total aroma volatiles, including monoterpenes, sesquiterpenes, aromatics, norisoprenoids, alcohols, and esters in the pulp of mango fruit (Lalel et al., 2003b). Lalel et al. (2003b) also found that MeJA increased the respiration rate at the beginning of ripening but did not affect the respiration rate at the climacteric stage in mango. In contrast, in apples, MeJA reduced the respiration rate during the later stages of storage (Ding et al., 2024).

### Aminocyclopropane carboxylic acid

Aminocyclopropane carboxylic acid (ACC) is a direct precursor of ethylene. Although it was first identified in plants in the 1950s (Burroughs, 1957), recent evidence has shown that ACC functions as a signaling molecule in plants independently of ethylene (Polko and Kieber, 2019). ACC affects early vegetative development and root elongation even when ethylene signaling is blocked by 1-MCP (Tsang et al., 2011; Vanderstraeten et al., 2019). In tomato, ACC promotes pollen tube growth independently of ethylene receptors, as demonstrated using the ethylene-insensitive Never Ripe mutant and 1-MCP treatment (Althiab-Almasaud et al., 2021). ACC’s independent activity is also evident when ethylene biosynthesis is inhibited by AVG (Tsang et al., 2011; Althiab-Almasaud et al., 2021).

In *Arabidopsis*, the *ein3 eil1* mutant is completely insensitive to ethylene and shows no response to ACC (Alonso et al., 2003). However, Tsang et al. (2011) observed that short-term ACC treatment induced the shortening of trichoblasts in *ein3 eil1* mutants in *Arabidopsis* roots. This growth inhibition was absent when combined with α-(phenylethyl-2-one)-indole-3-acetic acid (PEO-IAA), an auxin receptor antagonist, indicating that ACC signaling inhibits root elongation independently of ethylene, and auxin signaling is necessary for ACC signaling. Additionally, previous research indicated that ACC synthase is upregulated earlier in fruit ripening compared to ACC oxidase (Sitrit et al., 1986), suggesting that ACC may play a role in initiating the ripening process independently of ethylene. Due to the close relationship between ACC and ethylene, the exact extent to which their signaling pathways overlap or diverge remains to be fully elucidated (**Figure 1**).

### Brassinosteroids

Brassinosteroids (BRs) are steroid plant hormones that play key roles in plant development and defense. Regulation of brassinosteroid signaling and biosynthesis pathways were reviewed by Ye et al. (2011). BRs significantly promotes ethylene-mediated fruit ripening by upregulating ethylene biosynthesis genes in various fruits such as tomato and persimmon. In tomatoes, treatment with brassinolide (BL), the most active BR, increases the expression of ethylene biosynthesis genes *ACS2*, *ACS4*, *ACO1*, *ACO4*, and the key carotenoid biosynthesis gene *PSY1* (Zhu et al., 2015). Delving deeper, the overexpression of the Brassinazole Resistant1 (*BZR1*) gene, a downstream transcription factor of BR signaling, in tomato enhances fruit lycopene content by directly activating the transcription of *ACO1*, *ACO3*, and *PSY1* genes (Sang et al., 2022; Meng et al., 2023). Conversely, knocking out *BZR1* inhibits ripening through transcriptome reprogramming at the onset of ripening (Meng et al., 2023).

The story doesn’t end there. Knocking out Brassinosteroid-Insensitive2 (*SlBIN2*), which acts as a negative regulator upstream of SlBZR1 in BR signaling, accelerates fruit ripening and boosts carotenoid accumulation (Meng et al., 2023). Additionally, overexpression of the BR synthesis gene Dwarf (DWF), which plays a key role in catalyzing bioactive BRs like castasterone (CS), results in accelerated fruit softening, increased lycopene synthesis, and elevated ethylene levels in DWF-OE transgenic tomato plants (Sang et al., 2022). Furthermore, treating tomato plants with exogenous BR or enhancing endogenous BR levels by overexpressing the BR biosynthetic gene *SlCYP90B3* leads to an ethylene production surge, propelling fruit ripening forward, while the application of 1-MCP stifles this carotenoid accumulation (Sang et al., 2022; Hu et al., 2020).

In persimmon, the application of the brassinosteroid 24-epibrassinolide (EBR) increases the transcripts of *DkACO2*, *DkACS1*, and *DkACS2*, and upregulates several cell wall-modifying genes like *DkPG1*, *DkPL1*, *DkPE2*, and *DkEG1*, while treatment with brassinazole (Brz), a BR biosynthesis inhibitor, delays ripening (He et al., 2018). These findings underscore the pivotal role of BRs in regulating fruit ripening by acting through ethylene biosynthesis and signaling pathways.

### Salicylic acid

Salicylic acid (SA), a phytohormone well-known for inducing immune responses to pathogen attacks in plants, also plays a crucial role in inhibiting fruit ripening processes. In bananas, SA treatment results in reduced sugar content, lower invertase activity, decreased respiration rate, and less fruit softening. This is particularly evident in the reduced activity of major cell wall-degrading enzymes such as cellulase, polygalacturonase, and xylanase (Srivastava and Dwivedi, 2000). Additionally, acetylsalicylic acid (ASA), a derivative of SA, has been found to inhibit the rise in LOX activity and superoxide free radical production (Zhang et al., 2003; Mo et al., 2008). The positive correlation between lipoxygenase (LOX) activity and ethylene production in fruit tissue has already been demonstrated (Marcelle, 1991). Moreover, ASA reduces ACS and ACO activities in kiwifruit, leading to decreased ethylene biosynthesis, thereby delaying ethylene production and ripening (Zhang et al., 2003). Aghdam et al. (2010) also demonstrated that MeSA, another derivative of SA, inhibits ethylene production, decreases total soluble solids (TSS), and prevents fruit flesh softening of kiwifruits, thereby maintaining firmness and ascorbic acid content during storage. SA biosynthesis and signaling mechanisms were reviewed by Peng et al. (2021).

A recent study on pears (*Pyrus pyrifolia* Nakai) a transcriptomic analysis of SA-treated fruits showed the downregulation of genes such as *XTH*, *PME*, *β-gal*, and *EXP*, which inhibits firmness loss and cell wall degradation (Zhang et al., 2023b)(**Figure 1**). Along the same vein, another study on pears revealed that SA downregulated the expression of ethylene-responsive EIN3a but upregulated it in response to ethylene, auxin, and glucose. SA treatment increased the levels of both free and conjugated SA while dramatically reducing ethylene and auxin (indole-3-acetic acid, IAA) content during fruit senescence (Xu et al., 2023).

The effect of SA can vary widely based on concentration and timing of application. For example, in tomatoes, Ding and Wang (2003) found that low concentrations of methyl salicylate (MeSA), such as 0.1 mM at the mature green stage and 0.01 mM at the breaker stage, enhanced color development, ethylene production, and respiration. However, a higher concentration (0.5 mM) of MeSA inhibited these processes by reducing *ACS2* and *ACS4* transcript accumulation and delaying *ACO1* accumulation (Ding and Wang, 2003). These findings highlight the nuanced role of SA and its derivatives in modulating fruit ripening, offering potential strategies for managing fruit quality and extending shelf life.

## Relationship between ethylene and pre-harvest fruit drop

The relationship between fruit ripening and pre-harvest drop is deeply rooted in enzymatic actions, genetic factors, and genomic adaptations. During the ripening of climacteric fruits, enzymes such as expansin, cellulase and PG become more active. These enzymes degrade the cell walls within the abscission zone- a specific layer of cells where the fruit attaches to the stem. The activity of these enzymes increases as the fruit approaches ripeness (Yu-fa et al., 1982; Kondo and Takahashi, 1989; Malladi, 2020). This increase is correlated with a higher incidence of fruit softening and pre-harvest drop (Bonghi et al., 1992; Wakasa et al., 2003; Li and Yuan, 2008). Expansins modulate the loosening of the cell wall by disrupting non-covalent bonds between cellulose and hemicellulose, allowing other enzymes to access and degrade these components more effectively (Cosgrove, 2000). PGs break down pectin, a polysaccharide that acts as a glue holding cells together. The degradation of pectin facilitates cell separation at the abscission zone, enhancing the likelihood of fruit drop (Hadfield et al., 1998; Bonnin and Pelloux, 2020). Cellulases hydrolyze cellulose, a major component of the cell wall. In the abscission zone, increased cellulase activity results in the degradation of cell wall cellulose (Abeles, 1969; Tonutti et al., 1995; Li et al., 2010; Wang et al., 2022c). This degradation leads to the weakening of the fruit’s attachment, facilitating its drop.

Ethylene acts as a key hormonal signal that initiates and regulates the complex biochemical and physiological changes associated with pre-harvest drop. It activates the enzymatic breakdown mechanisms at the abscission zone (Brady and Speirs, 1991; Brown, 1997). Ethylene serves as a key signal molecule in initiating the transcription of several genes like *PGs*, cellulases, expansins (Li and Yuan, 2008; Eccher et al., 2015; Sriskantharajah et al., 2021). In apple, the expression of *MdPG1*, *MdEG1* (Endo- β-1,4-Glucanase) and *MdEXP3* increases in the fruit cortex and abscission zone during ripening, with a higher *ERS/ETR* ratio at transcript level indicating increased sensitivity to ethylene (Wakasa et al., 2003; Cin et al., 2005; Li and Yuan, 2008; Li et al., 2010). Ethylene signaling also interacts with other hormonal pathways (e.g. auxin, JA, and ABA), which can modulate the expression of abscission-related genes (Li and Yuan, 2008; Tadiello et al., 2016; Bai et al., 2021; Hu et al., 2022; Wang et al., 2022a). These findings suggest that ethylene directly or indirectly promotes ripening related pre-harvest fruit drop in climacteric fruits.

The application of ethylene inhibitors through pre-harvest sprays has been shown to effectively reduce fruit drop in climacteric fruits such as apples, thereby extending the harvest window (Bangerth, 1978; Greene and Schupp, 2004). These inhibitors increase fruit removal force, and inhibit ethylene biosynthesis, which are all critical factors responsible for reducing fruit drop (Bangerth, 1978). Pre-harvest application of AVG, 1-MCP, and Naphthaleneacetic acid (NAA), whether used in combination or alone, has proven to reduce premature fruit drop (Bangerth, 1978; Yuan and Carbaugh, 2007; Villalobos-Acuña et al., 2010; Yildiz et al., 2012). The effectiveness of AVG applications is particularly influenced by the timing and concentration used. Studies have shown that lower concentrations (150-300 mg/L) and application at 1 month before commercial harvest date are more effective than applying earlier or later in reducing fruit drop (Greene and Schupp, 2004; Schupp and Greene, 2004; Ozturk et al., 2015). Liu et al. (2022) found that a single application of AVG applied three weeks before the anticipated harvest (WBAH) is more effective than applications made at 1 WBAH. Moreover, the combination of AVG+NAA accompanied by 1-MCP has been found to more effectively delay pre-harvest fruit drop compared to the use of AVG or NAA alone (Yuan and Li, 2008). This synergistic effect suggests that a strategic approach, utilizing multiple ethylene inhibitors, can enhance the mitigation of fruit drop.

Predicting fruit drop in the climacteric fruit is a continuous challenge due to the intricate maturation processes that involve various physiological changes. One primary indicator of fruit drop is the change in fruit firmness and texture, which directly impacts the pre-harvest fruit drop and postharvest life of the product (Infante, 2012; Arseneault and Cline, 2016). Since direct measurement of flesh firmness before harvest is destructive and impractical for large-scale application, non-destructive technologies have been developed (Fathizadeh et al., 2021). Remote sensing technology like laser doppler vibrometer is a promising tool for determining fruit firmness and for evaluations of maturity (Muramatsu et al., 2000). In particular, optical properties near the chlorophyll absorbance range, have proven to be highly correlated with the flesh firmness (Infante, 2012). The Index of Absorbance Difference (I_AD_) has also shown strong correlations with chlorophyll degradation and flesh firmness that allows for continuous monitoring of on-tree fruit ripening, facilitating accurate determination of the optimal harvest time (Ziosi et al., 2008b). In addition to firmness and color, biochemical markers such as starch and sugar concentrations play a vital role in predicting fruit drop. In fruits like apples, a high starch concentration in the early stages indicates immaturity, while elevated levels of soluble sugars signal readiness for harvest maturity (Smith et al., 1979; Reid et al., 1982; Brookfield et al., 1997). Moreover, the loss of xylem functionality has been identified as an indicator of preharvest fruit drop potential. Due to its lignification, xylem is believed to be the last tissue to break down in the fruit pedicel, leading to preharvest fruit drop (Larson et al., 2023). There is a linear decrease in xylem functionality with rising internal ethylene content, correlating with increased preharvest fruit drop and higher expression levels of genes such as *MdEG1* and *MdPG2* in the fruit pedicel (Larson et al., 2023).

## Commercial applications

Commercial applications of hormones or plant growth regulators (PGRs) are pivotal in optimizing the ripening of climacteric fruits in commercial settings. Strategic use of PGRs allows for precise control of the ripening process, thereby enhancing market quality, extending the harvest season and shelf life, and ensuring uniform ripening stages to effectively meet consumer demands.

### Ethylene-based management of ripening

Commercial growers utilize ethylene as a key tool in managing fruit ripening to meet market demands for ripe and high-quality produce. Ethylene is widely used in commercial settings to stimulate and induce the ripening of various fruits such as bananas, avocados, tomatoes, and mangos (Zhang et al., 2016). While ethylene itself does not initiate ripening in immature fruits, exposure to ethylene accelerates the onset of maturity, leading to earlier ripening (Kevany et al., 2007). The application of ethylene-releasing agents like ethephon has been shown to effectively accelerate ripening, particular by increasing skin color and TSS (**Table 1**). This method allows growers to manage the ripening process efficiently, ensuring that fruits are ready for market at the desired stage of ripeness.

**Table 1:** Use of different plant growth regulators (PGRs) on modulating fruit ripening.

Hormone/PGR	Active ingredient	Conc. (ppm)	Crop	Ripening traits	Reference(s)
Ethylene/Ethephon	2-chloroethyl-phosphonic acid	500	Mango	Increased peel color	(Chen et al., 2022)
		500	Saskatoon	Increased fruit skin color	(McGarry et al., 2005)
		500	Banana	Higher TSS (Total Soluble Solids), lower firmness, increased peel color	(Lacap et al., 2021)
		500-1000	Avocado	Accelerated ripening and lower firmness	(Flores et al., 2021)
		150	Apple	Increased peel color	(Stover et al., 2003)
Ethylene Inhibitor/1-MCP	1-methylcyclopropene	400	Papaya	Delayed fruit softening	(Zhu et al., 2019)
		320-396	Apple	Reduced preharvest fruit drop and softening	(Yuan and Carbaugh, 2007; Yuan and Li, 2008)
		50	Pear	Reduced preharvest fruit drop and softening	(Villalobos-Acuña et al., 2010)
Ethylene Inhibitor/AVG	Aminoethoxyvinylglycine	125-150	Apple	Reduced preharvest fruit drop and reduced fruit color	(Wang and Dilley, 2001; Stover et al., 2003; Yuan and Carbaugh, 2007; Yuan and Li, 2008; Yildiz et al., 2012; Liu et al., 2022; Schultz et al., 2023)
Auxin/NAA	1-Naphthaleneacetic acid	93	Peach	Retained fruit firmness	(Li et al., 2021)
		ND^*^	Apple	Increased fruit color	(Dal Cin et al., 2008)
		10-20	Apple	Reduced fruit drop	(Stover et al., 2003; Arseneault and Cline, 2018)
Abscisic Acid/S-ABA	*S*-abscisic acid	1200	Date palm	Increased fruit color and TSS	(Elbar et al., 2023)
		132	Kiwifruit	Increased aroma	(Han et al., 2022)
		265	Banana	Increased fruit color	(Jiang et al., 2000)
		500	Fig	Increased peel color	(Lama et al., 2020)
Jasmonic Acid/(PDJ)	Prohydrojasmon/Propyl-3-oxo-2-pentylcyclo-pentylacetate	200	Apples	Increased fruit skin color	(Atay, 2015)
		100	Pear	Higher TSS, increased peel color	(Wang et al., 2020a)
		100	Peach	Increased fruit skin color and sucrose level	(Tang et al., 2022)
Jasmonic Acid/MeJA	Methyl jasmonate/Methyl cis-jasmonate	180	Peach	Increased fruit skin color and sucrose level	(Tang et al., 2022)
		1120	Apple	Increased fruit skin color	(Shafiq et al., 2013)

^*^ND, Not Determined.

To delay ripening and mitigate preharvest fruit drop, growers frequently utilize ethylene inhibitors. These inhibitors function either by delaying maturity, as seen with AVG and 1-MCP, or by inhibiting the production of cell hydrolysis enzymes in the fruit pedicel, such as NAA (Larson et al., 2023). Specifically, AVG and 1-MCP are employed to reduce preharvest fruit drop and delay fruit softening and harvesting, particularly in apples (**Table 1**). The application of 1-MCP followed by low-temperature storage (8-10°C) effectively extends the shelf life of various fruits, including mangoes, tomatoes, and plums, while minimizing economic losses during storage (Taye et al., 2019; Xu et al., 2020; Fayek et al., 2022). A significant advantage of ethylene biosynthesis inhibitors is their reversibility through exogenous ethylene treatment. For example, AVG application maintains higher flesh firmness, greener peel color, and reduced ethylene production (Steffens et al., 2006; Kang et al., 2007). However, AVG can also impede red color development in apples (Steffens et al., 2006). To counteract this, combining AVG with ethephon enhances red color development without sacrificing the maturation-delaying benefits of AVG. This combined treatment improves color development while preserving superior fruit quality during storage compared to untreated apples (Wang and Dilley, 2001; Steffens et al., 2006; Kang et al., 2007).

Similarly, the combined application of 1-MCP and ethephon has shown promise in extending the shelf life of bananas while maintaining normal ripening. Studies have demonstrated that 1-MCP (400 nL/L) combined with ethephon (50 μL/L) prolongs green-life during cold storage and induces uniform ripening during shelf-life (Satekge and Magwaza, 2020). This treatment effectively delays ripening without the uneven ripening often associated with 1-MCP alone (Botondi et al., 2014). Fruit size also influences 1-MCP efficacy, with medium and large fruit responding better to the treatment than small fruit. However, the combined 1-MCP and ethephon treatment shows consistent results across all fruit sizes, suggesting its potential as a beneficial postharvest treatment for bananas (Satekge and Magwaza, 2020).

The benefits of using ethylene inhibitors come with some trade-offs. For instance, while AVG treatments have been effective in delaying preharvest fruit drop, they have consistently been associated with a reduction in red skin color in apples (Wang and Dilley, 2001; Greene, 2005; Steffens et al., 2006; Liu et al., 2022). This reduction in red coloration due to AVG treatments can compromise the visual appeal of fruits, potentially leading to a decrease in market value. Similarly, application of 1-MCP for longer time increases storage flesh disorders. For instance, while short-term 1-MCP treatment delays ripening in papaya, long-term treatment can lead to ripening disorders, including a “rubbery” texture (Zhu et al., 2019). This disorder is associated with accelerated lignin accumulation and delayed cellulose degradation during ripening (Zhu et al., 2019).

In addition to AVG and 1-MCP, hydrogen sulfide (H_2_S) has emerged as a novel gaseous signaling molecule with the capacity to delay fruit ripening. It plays a complex regulatory role, often acting as an antagonist to ethylene, thereby slowing or inhibiting the ripening process (Yao et al., 2020). For example, H_2_S has been shown to reduce the activity of ACC oxidase. Specifically, treatments with H_2_S have been reported to suppress the expression of *SlACO1*, *SlACO3*, and *SlACO4* in tomatoes (Hu et al., 2019). Additionally, H_2_S may modulate ethylene receptors through post-translational modifications, such as S-sulfhydration, which reduces their sensitivity to ethylene, thereby weakening the signaling cascade that initiates ripening (Aroca et al., 2015; Ge et al., 2017; Jia et al., 2018). Another critical function of H_2_S in fruit ripening involves its capacity to scavenge reactive oxygen species (ROS), helping to maintain fruit quality post-harvest by preserving cellular integrity and delaying senescence. This has been observed in apples, tomatoes, and kiwifruits (Gao et al., 2013; Zheng et al., 2016; Yao et al., 2018; Ziogas et al., 2018).

### Use of JA and ABA for enhancing desired traits

In addition to ethylene, other hormones such as JA and ABA have been identified as key regulators of fruit ripening related traits including fruit coloration and anthocyanin accumulation. For instance, the JA derivative PDJ was found to significantly enhances color development in apples and other fruits by promoting anthocyanin accumulation. Specifically, PDJ application fosters the formation of red color in apples, pears, and peaches (Atay, 2015; Wang et al., 2020a; Tang et al., 2022). In apples, PDJ treatment is most efficacious when administered approximately two weeks before harvest, although the optimal timing may vary slightly among different cultivars (Atay, 2015). Notably, endogenous JA concentrations in apple pulp are high during the early stages of growth, decrease as the fruit develops, and then rise again during maturation (Kondo et al., 2000). In peaches, treatment with MeJA or PDJ positively influences red color formation due to an approximate 120% increase in anthocyanin accumulation. This enhancement is attributed to increased enzyme activities and elevated transcript levels of genes involved in anthocyanin biosynthesis induced by MeJA or PDJ (Tang et al., 2022). Similarly, Preharvest PDJ treatments markedly improve color development in red pears without adversely affecting other quality parameters such as total soluble solids and fruit acidity. PDJ treatments, particularly at a concentration of 100 mg/L, enhance the levels of anthocyanins and flavonols in the peel, while the concentrations of hydroxycinnamates and flavanols decrease (Wang et al., 2020a).

The levels of cyanidin 3-O-glucoside and cyanidin 3-O-rutinoside, the primary anthocyanins in fig fruit, increase with ABA treatment, while the expression of anthocyanin biosynthesis genes is downregulated by the ABA inhibitors nordihydroguaiaretic acid (NDGA) and fluridone (Lama et al., 2020). In mangoes, ABA treatment results in a yellower flesh color and upregulation of the carotenoid biosynthesis gene *MiPSY*, whereas fluridone inhibits carotenoid accumulation (Wu et al., 2022). Similarly, exogenous ABA accelerates carotenoid accumulation and chlorophyll degradation in cherry tomatoes (Wu et al., 2018b). Additionally, ABA application induces color change and softening in bananas, enhancing their visual appeal (Jiang et al., 2000). When ABA-treated bananas (265 ppm) were exposed to ethylene (100 ppm) for 24 hours, there was a notable increase in ethylene production and respiration, which further enhanced skin color changes and fruit softening beyond the effects of ethylene alone (Jiang et al., 2000).

ABA treatment also significantly promotes starch degradation and increases levels of soluble sugars (glucose and sucrose) in apples, accompanied by the induction of amylase genes and genes responsible for glucose and sucrose transport (Ma et al., 2017). Moreover, ABA treatment elevates the TSS content in mangoes, an effect inhibited by fluridone (Wu et al., 2022). In peaches, ABA maintains soluble sugar content during cold storage by enhancing the activities of sucrose phosphate synthase and sucrose synthase (Zhao et al., 2022).

### Auxin applications in ripening control

Auxin and its inhibitors hold significant practical implications for growers and the commercial sector in modifying fruit ripening traits. Understanding the interplay between auxin and ethylene allows growers to manipulate the ripening process, thereby extending the harvest season and enhancing both fruit quality and shelf life. The application of synthetic auxin or auxin-like compounds such as NAA has proven effective in delaying ripening in climacteric fruits, underscoring the utility of auxin manipulation. Research shows that NAA applications can significantly reduce preharvest fruit drop in apples, extending fruit retention on trees by approximately 14 days (Rehman et al., 2017). However, alternative treatments like AVG could be more effective than NAA in preventing fruit drop and delaying maturity (Fallahi, 2007). The effectiveness of reducing fruit drop by applying NAA could also be heightened when used in combination with AVG, particularly when applied 2-3 weeks before harvest (Yuan and Carbaugh, 2007; Arseneault and Cline, 2018).

NAA treatments have also been found to maintain Apple fruit firmness at harvest compared to untreated fruits (Ozturk et al., 2019). However, during extended cold storage, NAA-treated fruits may exhibit reduced flesh firmness (Ozturk et al., 2019). Additionally, NAA applications can enhance fruit color development, as indicated by decreased L* and hue angle values during storage (Ozturk et al., 2019). While NAA treatments may slightly increase fruit weight in later harvests compared to other treatments (Fallahi, 2007), they generally result in lower soluble solids content (SSC) (Ozturk et al., 2019). Furthermore, NAA-treated fruits tend to experience faster starch degradation (Ozturk et al., 2019).

## Future prospects

The future of fruit ripening management promises transformative advancements in our comprehension and control of this complex phenomenon. While the pivotal role of ethylene in climacteric fruit color change is well-documented, emerging research must probe the nuanced interactions between ethylene and other hormones and signaling molecules. For instance, investigating how JA, ABA, and auxin collaborate with ethylene to regulate ripening traits could unveil new dimensions in fruit development. Additionally, understanding these hormones’ multifaceted interactions with ethylene encompassing plant-microbe interactions, abiotic stress responses, and developmental cues may facilitate more optimized conditions that reconcile fruit ripening with other vital pathways. For example, auxin’s influence on xylem differentiation and tissue flexibility could be leveraged to mitigate physiological disorders, such as bitter pit in apples, that are manifested during ripening and post-storage (Griffith and Einhorn, 2023). However, the use of synthetic auxins like NAA might inadvertently affect ripening speed and fruit drop. Similarly, investigating the synergistic relationship between ethylene and JA and the antagonistic relationship between JA and SA in plant defense mechanisms against necrotrophic and biotrophic pathogens, respectively, (Phuong et al., 2020) alongside the above-mentioned roles of these hormones and their synthetic analogs in fruit ripening, can pave the way for informed spray applications and pest management strategies that account for these intricate hormonal interactions. By understanding these complex cross-talks, we can develop targeted approaches to enhance plant resilience and optimize fruit quality.

A promising avenue for future research involves examining ACC beyond its ethylene-related functions. Unraveling how ACC operates as an independent signaling molecule may reveal novel pathways and mechanisms influencing ripening and other physiological processes, potentially leading to precise ripening control. Preliminary findings from our group (unpublished) suggest that combining ACC with AVG may more effectively balance pre-harvest drop control and color development in apples compared to using these PGRs individually. Nonetheless, AVG’s role as an ACS enzyme inhibitor necessitates further elucidation of the molecular and biochemical mechanisms that could explain the observed effects.

Examining environmental conditions, genetic determinants, and crop load is crucial for elucidating their effects on ethylene production and sensitivity. Such analysis could provide valuable insights into pre-harvest fruit abscission and overall yield. Research should focus on how varying crop loads influence ethylene dynamics and fruit retention mechanisms, potentially uncovering strategies to enhance yield. Although there is a well-established correlation between fruit ripening and pre-harvest fruit drop, the precise nature of this relationship especially concerning skin color development and starch conversion as predictive indicators of pre-harvest drop remains ambiguous. Moreover, despite extensive investigations into ethylene mutants regarding the uniform segregation of ethylene-dependent ripening traits, the interplay between ripening characteristics and fruit abscission represents a compelling research frontier with potentially significant practical implications.

Addressing these research areas could lead to the development of more effective and sustainable fruit ripening management strategies. Such advancements will enhance fruit quality, reduce post-harvest losses, and improve the efficiency of fruit production and distribution, ultimately fulfilling consumer demands and fortifying the resilience of the agricultural industry.
